# Bilateral Biconvex Frontal Chronic Subdural Hematoma Mimicking Extradural Hematoma

**DOI:** 10.4103/2006-8808.73625

**Published:** 2010

**Authors:** Amit Agrawal

**Affiliations:** *Department of Neurosurgery, Datta Meghe Institute of Medical Sciences, Sawangi (Meghe), Wardha, Maharashtra, India*

**Keywords:** Chronic subdural hematoma, extradural hematoma

## Abstract

Chronic subdural hematoma (CSDH) is one of the most common clinical entities encountered in daily neurosurgical practice. The advent of computed tomography (CT) has made a major impact on the radiological diagnosis of CSDH. Although unilateral chronic isodense subdural hematomas as a result of indirect signs of a space-occupying lesion are easily recognizable on CT, bilateral CSDH may cause considerable difficulty, particularly when it is biconvex in shape as discussed in the present case. A judicious use of magnetic resonance imaging will help in making the diagnosis and for the management of such lesions.

## INTRODUCTION

Chronic subdural hematoma (CSDH) is one of the most common clinical entities encountered in daily neurosurgical practice[1] CSDH is an encapsulated collection of old blood, mostly or totally liquefied and located between the dura mater and arachnoid.[2] We discuss the clinical and radiological findings in a case of bifrontal biconvex chronic subdural that was mimicking the extradural hematoma.

## CASE REPORT

**Figure 1:**
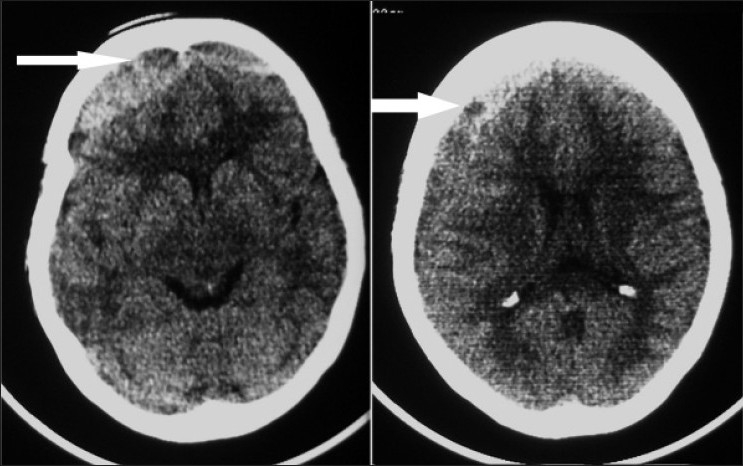
Computed tomogram without contrast, showing large bifrontal hyperdense convex-shaped collection (left), also associated crescent-shaped collection over right frontoparietal region (right) (note - both the frontal horns of lateral ventricles are widely displaced laterally and pushed posteriorly), also note inhomogeneous nature of the hematoma (a tendency of liquefaction of the blood on the right high parietal region-arrows)

A 56-year-old woman presented with the history of fall on her back 2 months back. She was apparently alright till one week back when she started with weakness of left upper and lower limb and mild headache. Her general and systemic examination was unremarkable. Higher mental functions were normal. She had mild bilateral papilloedema. There was grade IV/V weakness involving left upper and lower limb with exaggerated ipsilateral deep tendon reflexes and extensor plantar. There were no other neurological deficits. Her computed tomography (CT) scan showed bifrontal biconvex hyperdense (right more than left) with mass effect in the form of compression and wide splaying of both the frontal horns of the lateral ventricles [Figure 1 left].

**Figure 2:**
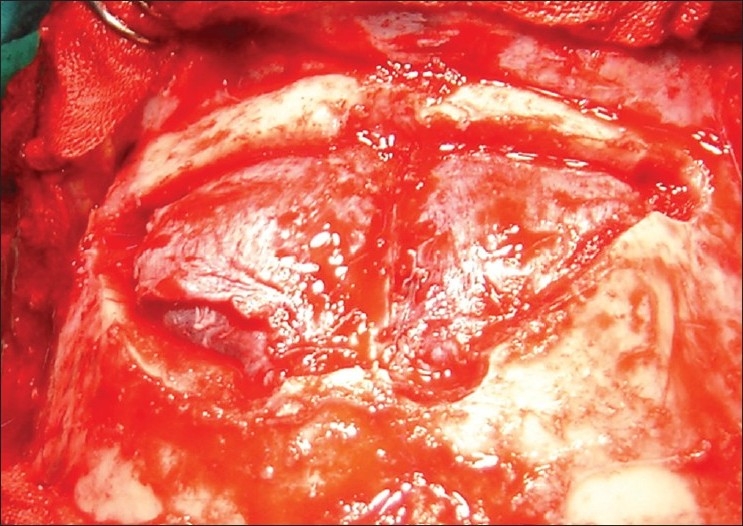
Intraoperatively, there was no extradural collection but associated bluish discoloration of dura

In higher sections, the lesion was becoming inhomogeneous (a tendency of liquefaction of the blood on the right high parietal region) and crescentic in shape [Figure 1 right]. Though there was a classical history suggestive of CSDH, in view of CT scan findings, a remote possibility of bifrontal extradural hematoma was also considered. As we suspected the diagnosis of bifrontal extradural hematoma, the patient underwent bifrontal craniotomy; however, there was no evidence of extradural hematoma but dura was tense and bluish in color [Figure 2]. Small openings in dura were made on both the sides and altered blood was evacuated from the subdural space. Patient is doing well after surgery.

## DISCUSSION

The advent of CT has made a major impact on the radiological diagnosis of CSDH and nowadays, most of the cases are diagnosed on cranial CT.[2,3] Although unilateral chronic isodense subdural hematomas as a result of indirect signs of a space-occupying lesion are easily recognizable on CT, bilateral CSDH may cause considerable difficulty.[4] A specific finding in CSDH is the displacement of the brain parenchyma away from the skull and the usual convex border appears flattened or even concave. Also, several other indirect features due to the displacement of the brain—for example, effacement of the sulci, compression of the ipsilateral ventricle leading to midline shift, deformity of the normal ventricular anatomy, and obliteration of the basal cisterns—could aid in the diagnosis.[5–7] Bilateral hematomas may lead to medial compression of both ventricles resulting in a narrow, slit-like elongated ventricle (the anterior horns sharply pointed and approaching one another so called ‘squeezed ventricle,’ ‘hare’s ears sign, or ‘rabbit’s ears’).[4–7] This sign together with clinical data is always suspicious of chronic bilateral subdural hematomas.[4] All these signs may hold true for the lesions that are placed supereolaterally. But if the lesion is placed more anteriorly and medially, hyperdense in intensity and enclosed in thick capsule, it may look biconvex in shape and can mimic extradural hematoma. This location of the lesion will also displace the frontal horns of the lateral ventricles laterally than medially, as in the present case. To avoid this confusion, if available, magnetic resonance imaging (MRI) would be better than CT in identifying these lesions.[3,8–10] Based on MRI, CSDHs can be classified into five types on both T(1)- and T(2)-weighted images: low, high, and mixed intensity, isointensity, and layered.[3] Usually, CSDHs are hyperintense on both T1- and T2-weighted MRI (the T1 values of CSDHs are significantly shorter than gray matter values and significantly longer than white matter values and the T2 values are significantly longer than both gray matter and white matter values).[3,10] However, it can be iso- or hypointense on T1-weighted images in some cases.[10] Even though MRI has advantages, CT remains the procedure of choice in the acute setting because of shorter examination time, which is important in acutely ill patients, reliability in identifying other lesions.[2] In addition to the CT scan, a judicious use of MRI in present case would have helped in making a correct diagnosis and also in the surgical management of such lesions.
